# Undifferentiated embryonal sarcoma of the liver in a middle-aged adult with systemic lupus erythematosus

**DOI:** 10.1186/1477-7819-11-244

**Published:** 2013-09-28

**Authors:** Changjun Jia, Wei Zhao, Chaoliu Dai, Xinlu Wang, Xianmin Bu, Songlin Peng, Feng Xu, Yongqing Xu, Yang Zhao

**Affiliations:** 1 Department of General Surgery, Shengjing Hospital, China Medical University, Shenyang 110004 Liaoning Province, P R China; 2 Department of Pathology, Shengjing Hospital, China Medical University, Shenyang 110004, Liaoning Province, P R China; 3 Department of Ultrasound Medicine, Shengjing Hospital, China Medical University, Shenyang 110004, Liaoning Province, P R China

**Keywords:** Undifferentiated embryonal sarcoma, Liver, Systemic lupus erythematosus, Diagnosis

## Abstract

Adult primary undifferentiated embryonal sarcoma of the liver (UESL) is a rare disease. While the etiology of UESL remains largely unknown, association with systemic inflammatory disorders has been observed. Here, we report a case of UESL in a 46-year-old woman with systemic lupus erythematosus (SLE) and without chronic hepatitis or liver cirrhosis. Systematic review of the publicly available English language medical literature identified only 27 cases of UESL in patients aged >45 years and none with SLE. Our patient presented with abdominal pain and had a 2-year history of SLE. Abdominal ultrasonography and enhanced computed tomography revealed a solid mass in the right lobe of the liver. Presumptive diagnosis of atypical hepatocellular carcinoma was made and the patient was treated with segmentectomy of S5 and S4a and cholecystectomy. The final diagnosis of UESL was made according to the pathology results. Since SLE patients may be at increased risk of malignancy, it is possible that the SLE pathogenesis may have contributed to the development of UESL in our patient. According to this case, UESL should be considered when SLE patients present with hepatic space-occupying lesions.

## Background

Undifferentiated (embryonal) sarcoma of the liver (UESL; also known as malignant mesenchymoma of the liver) is a malignant hepatic tumor that is composed of undifferentiated mesenchymal cells. The majority of the reported cases occurred in children between the ages of 6 and 15 years [1,2], but occasional diagnosis of UESL in adult patients (defined in the literature as aged >15 years) have also been reported [3]. UESL in patients aged 45 years or older is extremely rare, and only 27 such cases have been reported in the English language medical literature up to 2012. With the exception of two cases that are not well documented, these cases are summarized in Table 1[3-24]. The etiology of this rare disease remains largely unknown, especially in adult patients. However, a potential association with inflammatory disease has been suggested based upon some cases with co-morbidities, such as CREST syndrome, malaria, community-acquired pneumonia, cancer-related chemotherapy, and multiple sclerosis [6,8,12,15,22].

**Table 1:** Summary of the clinical features reported for undifferentiated liver embryonal sarcoma in patients aged over 45 years

**No.**	**Reference**	**Publication year**	**Sex**	**Age(years)**	**Symptoms and signs**	**Laboratory findings**	**Location**	**Size (cm)**	**Surgery**	**Adjuvant therapy**	**Recurrence**	**Follow-up, duration**	**Co-morbidity or past history**
1	Mattila et al. [4]	1974	F	68	RUQA pain, hepatomegaly	↑ALP, LDH, Bilirubin	R + L	20 × 18 × 15	Rx ext hep	None	Yes	DOD, 5 months	None
2	Tanner et al. [5]	1978	F	66	RUQA pain, vomiting, diarrhea, weight loss, fever, hepatomegaly	↑ALP, GGT	R	NA	HA ligation + pump	5-FU	-	AWD, 8 months	CHD
3	Chang et al. [6]	1983	F	55	RUQA pain, weight loss, diarrhea, hepatomegaly	↑ALP	L	10 × 10 × 8	Left lob	CAV	No	ANED, 12 months	CREST syndrome
4	Ellis et al. [7]	1983	F	86	RUQA pain, RUQA mass	Normal	R	18 × 12 × 12	Tumorectomy	None	Yes	DOD, 2 months	None
5	Forbes et al. [8]	1987	M	69	RUQA pain, nausea, weight loss, general malaise	Normal	NA	NA	None	RT	-	DOD, 10 months	Malaria and resection for colonic carcinoma
6	Forbes et al. [8]	1987	F	49	RUQA pain, nausea, weight loss, general malaise	NA	NA	NA	None	None	-	DOD	None
7	Grazi et al. [9]	1996	F	60	RUQA mass, dyspepsia	Normal	R	22	Rx hep	None	Yes	DOD, 10 months	NA
8	Nishio et al.[3]	2003	F	49	RUQA pain, general fatigue	NA	R	14 × 8 × 8	Rx lob	Adm + Dtic	Yes	DOD, 29 months	None
9	Nishio et al. [3]	2003	M	62	RUQA pain	NA	L	10 × 9 × 7	Left lob	None	No	ANED, 10 months	NA
10	Lepreux et al. [10]	2005	F	51	RUQA pain, dyspnea, edema of the lower limbs	Normal	R	18 × 16	Liver biopsy	CAV	NA	DOD, 2 months (died from septic shock and acute pneumopathy)	NA
11	Lepreux et al. [10]	2005	F	49	RUQA pain, increased abdominal girth	Normal	L	15 × 13	Left lob	MAID, RT	No	ANED, 6 months	NA
12	Agaram et al. [11]	2006	F	50	NA	NA	NA	17	NA	NA	No	ANED, 8 months	NA
13	Scudiere et al. [12]	2006	F	51	NA	↑ALT, AST	R	23 × 16 × 7	Rx lob	NA	NA	NA	Community-acquired pneumonia
14	Ma et al. [13]	2008	F	61	RUQA pain	Normal	R	12 × 9 × 8	Rx hep	None	NA	DOD, 8 months	None
15	Yang et al. [14]	2009	M	46	Upper abdominal pain, fever, mass, hepatomegaly	↑ALT, GGT, AFP HBeAb(+), HBcAb(+)	R	6 × 6 × 5	Rx hep	NA	NA	NA	Hepatitis B cirrhosis and HCC in the left lobe
16	Yang et al. [14]	2009	M	54	Upper abdominal pain, fever, mass, hepatomegaly	↑ALT, AST, GGT	R	13 × 12 × 12	Rx hep	NA	NA	NA	None
17	Kullar et al. [15]	2009	F	52	RUQA pain, RUQA mass	↑ALP	R	18 × 12 × 8	Rx ext hep	Adm + Ifs	Yes	DOD, 12 months	Chemotherapy for ovarian cancer
18	Yoon et al. [16]	2010	F	53	Abdominal discomfort	Normal	R	13 × 12 × 8	Rx hep	None	No	ANED, 6 months	None
19	Massani et al. [17]	2010	F	47	Upper abdominal pain	Normal	R	8	Rx hep	Epi + Ifs; TACE with Epi	Yes	AWD, 29 months	None
20	Li et al. [18]	2010	M	63	RUQA pain, fever	↑ALP	R	20 × 15 × 15	Rx hep	NA	Yes	DOD, 18 months	None
21	Li et al. [18]	2010	M	56	RUQA pain	↑ALP, ALT, AST, GGT	R	13 × 12	Rx hep	TACE with lipiodol, Epi, hydroxycamptothecin	Yes	DOD, 28 months	None
22	Gasljevic et al. [19]	2011	F	58	Asymptomatic	NA	R	11 × 8	Rx lob	Cysplatin + 5-FU + Vcr	No	DOD, 10 months (died from unrelated cause, B-ALL)	None
23	Kim et al. [20]	2011	F	47	Abdominal mass	↑CA-125	L	12 × 10	Left hep	MAID, RT	Yes	AWD, 48 months	None
24	Legou et al. [21]	2012	F	61	Upper abdominal pain, RUQA mass	↑ALP, ALT, AST	L	27 × 22 × 10	Tumorectomy	NA	NA	NA	None
25	Tanaka et al. [22]	2012	F	53	Asymptomatic	Liver function tests were slightly abnormal	R	12 × 11 × 7	Rx lob	None	Yes	DOD, 36 months	Multiple sclerosis
26	Tucker et al. [23]	2012	F	74	Abdominal pain and fullness, back pain	Normal	L	18 × 15 × 13	Left hep	None	No	ANED, 9 months	NA
27	Lightfood et al. [24]	2012	F	78	Abdominal mass	Normal	R	16	Partial right hepatectomy	None	No	ANED, 6 months	NA
28	This report	2013	F	49	Upper abdominal pain	Normal	R	7 × 6 × 5	Rx lob	Traditional Chinese medicine orally	Yes	DOD, 12 months	SLE

*5-FU*, 5-fluouracil; *Adm*, doxorubicin; *AFP*, α-fetoprotein; *ALP*, alkaline phosphatase; *ALT*, alanine aminotransferase; *ANED*, alive with no evidence of disease; *AST*, aspartate aminotransferase; *AWD*, alive with disease; *B-ALL*, B-lymphoblastic leukemia; *CA-125*, carbohydrate antigen 125; *CAV*, cyclophosphamide + Adm + Vcr; *CHD*, coronary heart of disease; *CREST syndrome*, calcinosis, Raynaud phenomenon, esophageal dysmotility, sclerodactyly, and telangiectasia syndrome; *DOD*, dead of disease-related cause; *Dtic*, decarbazine; *Epi*, epirubicin; *F*, female; *GGT*, γ-glutamyl transpeptidase; *HA*, hepatic artery; *HCC*, hepatocellular carcinoma; *Ifs*, ifosfamide; *L*, left; *Left hep*, left hepatectomy; *Left lob*, left lobectomy; *M*, male; *MAID*, Adm + Ifs + Dtic + methylprednisolone; *NA*, not available; *R*, right; *RT*, radiation therapy; *RUQA*, right upper quadrant abdominal; Rx ext hep, extended right hepatectomy; Rx hep, right hepatectomy; Rx lob, right lobectomy; *SLE*, systemic lupus erythematosus; *TACE*, transcatheter arterial chemoembolization; *Vcr*, vincristine.

SLE is a relatively common chronic inflammatory disease that affects multiple organs but only rarely causes serious liver impairment. While SLE has not been previously associated with UESL, it is associated with an increased risk of malignancy, particularly with hepatic cavernous hemangioma, lymphoma, and hepatocellular carcinoma (HCC) [25-27]. Herein, we report a new case of UESL that occurred in a middle-aged female with SLE. The patient had a medical history of steroid hormone therapy to treat the SLE but no medications or co-morbidities related to cancer or liver damage. To the best of our knowledge, this is the first report of a concomitant case of UESL and SLE. No distinctive symptoms, tumor markers, or imaging features were found and UESL diagnosis was based on surgical pathology.

## Case presentation

In January 2010, a 46-year-old woman presented with a complaint of idiopathic upper abdominal pain that had lasted for about 20 days. Self-report of personal and medical history indicated no recent weight loss, no symptoms or diagnoses of hepatitis or other liver diseases, no drug abuse, and no specific family history of cancer. However, the patient revealed a diagnosis of SLE from 2 years prior, since which she had remained on a prednisone (PSL) regimen of 10 mg/day.

Physical examination on admission showed that the patient was well nourished and had no signs of jaundice, icteric sclera, spider naevi, or palmar erythema. The cardiopulmonary examination was unremarkable. Abdominal examination revealed it to be soft and without tenderness or palpable mass. However, palpation of the hepatic region elicited pain, so that no further examination could be performed below the xiphoid and the right lower costal margin of the liver. Laboratory tests showed blood cell counts, liver function, renal function, and coagulation time within the normal ranges. Tests for hepatitis B and C viruses were negative. Tests for the tumor markers carcinoembryonic antigen (CEA; general cancer), α-fetoprotein (AFP; HCC), and cancer antigen 19–9 (CA19-9; gastrointestinal cancer) were normal. The test for the autoimmune disorder marker antinuclear antibody (ANA) was positive at a titer of 1:320. Tests for deficiencies in the complement system indicated normal activity, with C_3_ being 1.08 g/L(0.74-1.4 g/L) and C_4_ being 0.186 g/L(0.12-0.36 g/L). Likewise, quantitative immunoglobulin (Ig) testing indicated that the immune system status was normal, as indicated by IgA at 3.86 g/L(0.97-3.2 g/L), IgM at 0.34 g/L(0.4-1.59 g/L), and IgG at 10.10 g/L(6.95-15.15 g/L). No antibodies against double-stranded DNA, ribonucleoprotein, Smith antigen, SSA/Ro, SSB/La, SCl-70, or JO-1 were detected.

**Figure 1:**
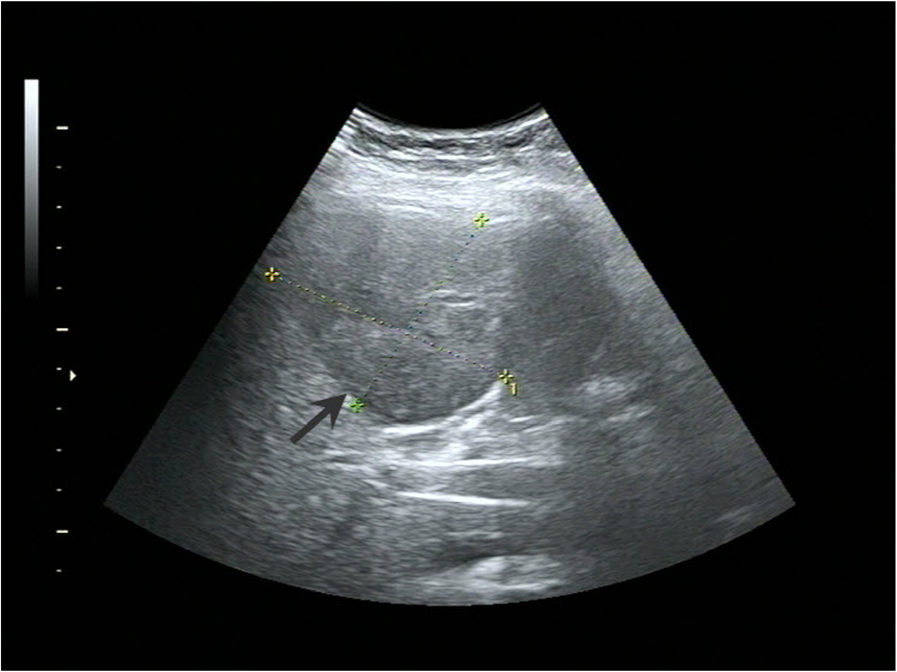
Transverse ultrasound scan of the upper abdomen showing a non-homogeneous, iso-hypoechoic and solid lesion in the right lobe of the liver.

**Figure 2:**
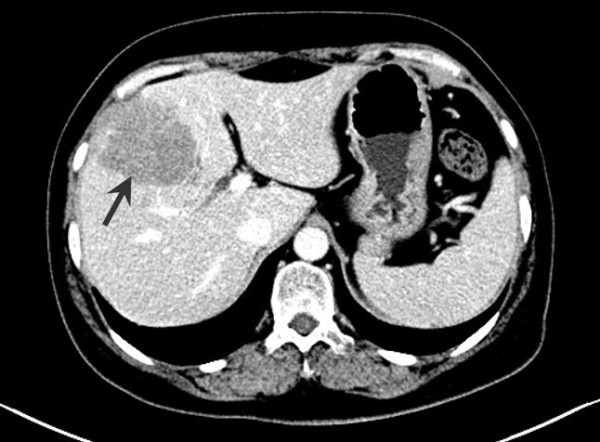
**Contrast-enhanced CT axial section of the abdomen showing ahypodense lesion in the right lobe of the liver.** The lesion is differentiated from the surrounding hepatic tissue by lower enhancement.

Abdominal ultrasound examination revealed a 6.3 × 5.5 cm solid lesion in the right lobe of the liver. The lesion showed inhomogeneous echogenicity, with iso-and hypo-echoicareas, and had indistinct boundaries (Figure 1). Abdominal contrast-enhanced computed tomography (CT) revealed an irregularly shaped space-occupying lesion in the right anterior lobe of the liver that measured 6.0 × 5.0 cm. During the unenhanced phase, the lesion appeared slightly hypodense (CT value of 42 HU); during the hepatic arterial phase, the enhancement of the lesion was obviously lower than that of the surrounding tissues (Figure 2). Delayed scanning showed that the enhancement of the lesion did gradually increase but always remained lower than that of the surrounding tissues. The overall imaging characteristics of the lesion were non-specific and the differential diagnoses that were considered included secondary hepatic neoplasm, biliary neoplasm, and atypical hepatocellular carcinoma. Colonoscopy and gastroscopy examinations were performed and yielded normal findings. Similarly, the chest X-ray and whole body bone scan was normal.

Based upon these collected findings, the initial diagnosis was primary liver neoplasm. As such, segmentectomy of S5 and S4a and cholecystectomy were performed under general anesthesia. During the operation, a solid mass with 6 cm diameter was located in the right anterior lobe of the liver. Exploration of the left hepatic lobe and abdominal cavity found a normal structure and no other tumors. Finally, a partial hepatectomy was carried out to remove the entire lesion from the right lobe, which included a sufficient portion of the normal liver tissue to help ensure complete resection; the resection status was confirmed as R0.

**Figure 3:**
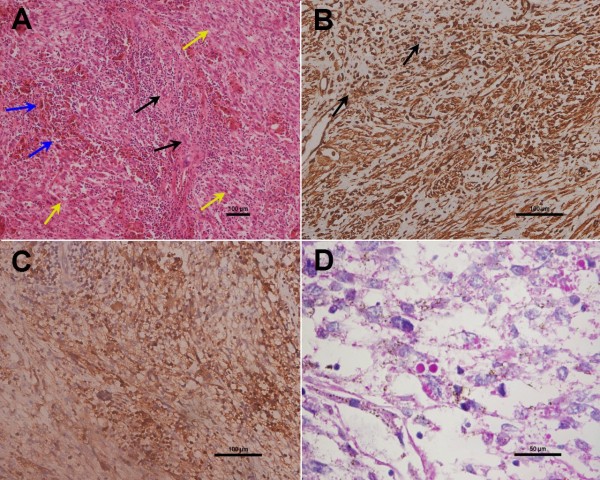
**Histological findings and immunohistochemical expression in UESL. (A)** H&E stained sections showing the tumor cells as spindle- or polygonal-shaped with fascicular arrangement (yellow arrows). In addition, multinucleate giant cells (blue arrows), remnants of small bile ducts (black arrows), and chronic inflammatory cells infiltration are shown in the tumor tissue (magnification: 100×). **(B, C)** Immunohistochemistry analysis of UESL showing positivity for **(B)** vimentin and **(C)** AAT (magnification: 200×). In **(B)**, black arrows indicate the coutnerstained cell nuclei. **(D)** Cells containing eosinophilic cytoplasmic granules staining positive for PAS are shown (magnification: 400×).

Histological analysis of the resection specimen defined the 6.5 × 6 × 5 cm lesion as well circumscribed, lobulated and soft, with yellowish-brown and grayish-white coloration. Observation of a gross cross-sectional area of the lesion showed dark red coloration and a small variegated portion. Microscopic examination showed that the tumor cells were spindle- or polygonal-shaped, arranged in a fascicular pattern, and distributed diffusely throughout the mass (yellow arrows); in addition, multinucleate giant cells (blue arrows), remnants of small bile ducts (black arrows), and chronic inflammatory cell infiltration were also detected in the tumor tissue (Figure 3A). Immunohistochemical staining for cancer markers defined the tumor as positive for vimentin, alpha-1-antitrypsin (AAT), and periodic acid-Schiff (PAS) (Figure 3B, 3C, 3D), but negative for cytokeratin (CK)AE1/AE3, S-100, epithelial membrane antigen (EMA), alpha-smooth muscle actin (SMA), myogeinc differentiation 1 (Myo D1), CK8/18, hepatocyte paraffin 1, human melanoma black-45, melan-A, CD117, synaptophysin, anaplastic lymphoma kinase, CD21, CD35, CD23, and CD34. The tumor cells were positive for vimentin and AAT, which indicated that the tumor was composed of undifferentiated mensenchymal cells. The immunoreactive negative findings for CK, S-100, EMA, alpha-SMA, and MyoD1 indicated there was no epithelium, nervous, smooth muscle, or striate muscle differentiation. The immunoreactive negative findings for the other markers excluded hepatocellular carcinoma, melanoma, gastrointestinal stromal tumor, neuroendocrine tumor, lymphoma, and dendritic cell tumor. The final diagnosis based on these pathology findings was UESL.

The patient recovered from surgery without complication and was discharged on day 12 postsurgery. No adjuvant chemotherapy or transcatheter arterial chemoembolization (TACE) was performed according to the patient’s insistence on only taking oral traditional Chinese medicines. At the 6-month follow-up, an unresectable tumor was detected, but the patient again refused palliative therapy (chemotherapy or TACE). The patient died 1 year later.

## Discussion

As a rare disease with few case reports in the literature, the etiology, cellular origin, and carcinogenic mechanisms of UESL remain unclear. In most cases of UESL, no pre-existing liver disease is detected [28]; however, some of the middle-aged and elderly patients diagnosed with UESL also suffered from multiple sclerosis [22], concomitant coronary heart disease [5], CREST syndrome [6], or community-acquired pneumonia [12]. In addition, some of these patients have a past history of malaria [8] or cancer, including patients who have undergone resection for colonic carcinoma [8] or chemotherapy for ovarian cancer [15]. Interestingly, only one of the previously reported adult UESL cases aged >45 years had hepatitis B cirrhosis and HCC in the left lobe (Table 1), which may have contributed to the development of the UESL.

The new UESL adult case described herein was unique for the concomitant SLE disease status and history of steroid therapy. The clinical characteristics of SLE-associated liver disorders include hepatomegaly, jaundice, and elevated liver enzyme levels; it is unknown, however, whether these complications are directly induced by the SLE pathogenic process itself, the drugs used to treat SLE, or secondary thrombotic events induced by SLE [25-27]. Both benign (for example, hemangioma) and malignant lesions have been found in the livers of patients with SLE and may be related to a localized chronic inflammatory state. Unfortunately, these hepatic lesions may be difficult to distinguish radiographically, and it may be necessary to perform a biopsy to exclude a neoplasm diagnosis.

Review of the middle-aged and elderly UESL cases reveals some potential distinguishing characteristics from the UESL pediatric cases. Where the pediatric cases of UESL show equal distribution among boys and girls [28], there are more female cases among the middle-aged and elderly patients with a female-to-male ratio of 3.5:1 (Table 1). In addition, where the pediatric UESL cases often present with a large palpable mass with or without abdominal pain, the middle-aged and elderly patients often have non-specific symptoms and signs. The most frequently reported complaints in the older population are abdominal pain or discomfort and detection of an abdominal mass. However, in both the pediatric and older adult populations, the lesions tend to be located in the right lobe of the liver.

Further consideration of the adult cases in the literature indicated that most patients have normal laboratory results and partial increase in the level of serum alkaline phosphatase (ALP). Only the patients with concomitant hepatitis-related cirrhosis or HCC showed elevated serum AFP and another patient with elevated serum CA-125 [15,20]. According to the reported ultrasound findings for these patients, >80% of UESL are solid. However, CT and magnetic resonance imaging analyses revealed punctate regions that were identified as liquified necroses and blood clots in one patient [28]. In the new case described herein, ultrasound examination revealed an inhomogeneous iso-hypoechoic solid lesion and CT showed an avacular entity visibly different from the surrounding hepatic tissues.

The histopathological features of UESL are also slightly different between the pediatric patients and the middle-aged and elderly patients. Unlike the UESL of pediatric patients, that of older patients tends to have a less prominent myxoid component and show at least a partial smooth muscle phenotype [3]. Moreover, the older adult UESL are diffusely positive for vimentin and AAT, and focally positive for cytokeratin, desmin, SMA, muscle-specific actin, CD68, myoglobin, non-specific enolase, S-100, and CD34 [18]. It has been reported that >80% of the UESL tumor cells overexpress p53 and suggested that the p53 pathway may plays a role in the disease onset in older adult patients [10].

Unfortunately, the limited number of UESL patients has also hindered attempts to understand the pathogenesis of this potentially life-threatening disease and to develop preventive strategies and effective therapies. Prognosis of UESL remains poor, and the median survival is <1 year after diagnosis [28]. Currently, the most effective therapeutic intervention is complete surgical resection or liver transplantation plus pre- and/or postoperative systemic chemotherapy [29,30]. Indeed, there are reports of prolonged survival in UESL patients (both children and adults) with radical operation followed by adjuvant chemotherapy [2,29]. Recently, May et al. reported that five pediatric UESL patients treated with a uniform approach of resection followed by adjuvant chemotherapy (VAC therapy) and radiation (as indicated on a case-by-case basis) achieved disease-free status and that the median length of their first remission was 53 months [31]. TACE is another choice of adjuvant therapy for liver cancer patients. Although the therapeutic effect of a single TACE application did not achieve good results for resolving the hypovascularity of most UESL patients, a small case series report indicated that TACE after complete resection of UESL improved the survival time [18], suggesting that some UESL cases might be highly sensitive to TACE. Adding more cases to the published literature may improve our ability to diagnose and manage UESL.

## Conclusions

In conclusion, we report a case of primary UESL concomitant with SLE in a middle-aged female patient without established chronic liver disease. The patient had no specific symptoms and no distinguishing findings for tumor markers or cellular and tissue structures. Final diagnosis was made by surgical pathology findings, although the precise etiology of UESL in this SLE patient remains unknown. It is possible that the long-term steroid therapy and chronic immune disorder may have contributed to disease onset or promoted its progression.

## Consent

Written informed consent was obtained from the patient for publication of this case report and any accompanying images. A copy of the written consent is available for review by the Editor-in-Chief of this journal.

## Competing interests

The authors declare that they have no competing interests.

## Authors’ contributions

CJ, SP, FX, YX, and YZ prepared the manuscript and the literature search; CJ drafted the manuscript; CD and XB corrected and revised the manuscript; CD and CJ treated and observed the patient; XW provided the radiographic and ultrasound images; WZ performed the histopathological and immunohistochemical examinations. All authors read and approved the final manuscript.

## References

[B1] Stocker JT Ishak KG Undifferentiated (embryonal) sarcoma of the liver: report of 31 cases Cancer 1978 42 336 348 10.1002/1097-0142(197807)42:1<336::AID-CNCR2820420151>3.0.CO;2-V 208754

[B2] Lenze F Birkfellner T Lenz P Hussein K Länger F Kreipe H Domschke W Undifferentiated embryonal sarcoma of the liver in adults Cancer 2008 112 2274 2282 10.1002/cncr.23431 18361435

[B3] Nishio J Iwasaki H Sakashita N Haraoka S Isayama T Naito M Miyayama H Kikuchi M Undifferentiated (embryonal) sarcoma of the liver in middle-aged adults: smooth muscle differentiation determined by immunohistochemistry and electron microscopy Hum Pathol 2003 34 246 252 10.1053/hupa.2003.44 12673559

[B4] Mattila S Keskitalo E Makinem J Primary non-differentiated sarcoma of the liver: case report and review of the literature J Acta Chir Scand 1974 140 303 307 4837812

[B5] Tanner AR Bolton PM Powell LW Primary sarcoma of the liver: report of a case with excellent response to hepatic artery ligation and infusion chemotherapy Gastroenterology 1978 74 121 123 72700

[B6] Chang WW Agha FP Morgan WS Primary sarcoma of the liver in the adult Cancer 1983 51 1510 1517 10.1002/1097-0142(19830415)51:8<1510::AID-CNCR2820510826>3.0.CO;2-K 6681727

[B7] Ellis IO Cotton RE Primary malignant mesenchymal tumour of the liver in an elderly female Histopathology 1983 7 113 121 10.1111/j.1365-2559.1983.tb02221.x 6840705

[B8] Forbes A Portann B Johnson P Willianms R Hepatic sarcomas in adults: a review of 25 cases Gut 1987 28 668 674 10.1136/gut.28.6.668 3623214 PMC1433048

[B9] Grazi GL Gallucci A Masetti M Jovine E Fiorentino M Maziotti A Gozzetti G Surgical therapy for undifferentiated (embryonal) sarcomas of the liver in adults Am Surg 1996 62 901 906 8895710

[B10] Lepreux S Rebouissou S Le-Bail B Saric J Balabaud C Bloch B Martin-Négrier ML Zucman-Rossi J Bioulac-Sage P Mutation of TP53 gene is involved in carcinogenesis of hepatic undifferentiated (embryonal) sarcoma of the adult, in contrast with Wnt or telomerase pathways: an immuohistochemical study of three cases with genomic relation in two cases J Hepatol 2005 42 424 429 10.1016/j.jhep.2004.10.021 15710230

[B11] Agaram NP Baren A Antonescu CR Pediatric and adult hepatic embryonal sarcoma: a comparative ultrastructural study with morphologic correlations Ultrasturct pathol 2006 30 403 408 10.1080/01913120600854699 17182431

[B12] Scudiere JR Jakate S A 51-year-old woman with a liver mass: undifferentiated embryonal sarcoma of the liver Arch Pathol Lab Med 2006 130 e24 e26 16454576 10.5858/2006-130-e24-AYWWAL

[B13] Ma L Liu YP Geng CZ Tian ZH Wu GX Wang XL Undifferentiated embryonal sarcoma of liver in an old female: case report and review of the literature World J Gastroenterol 2008 14 7267 7270 10.3748/wjg.14.7267 19084947 PMC2776890

[B14] Yang L Chen LB Xiao J Han P Clinical features and spiral computed tomography analysis of undifferentiated embryonic liver sarcoma in adults J Dig Dis 2009 10 305 309 10.1111/j.1751-2980.2009.00400.x 19906110

[B15] Kullar P Stonard C Jamieson N Huquet E Praseedom R Jah A Primary hepatic embryonal sarcoma masquerading as metastatic ovarian cancer World J Surg Oncol 2009 7 55 10.1186/1477-7819-7-55 19549298 PMC2705365

[B16] Yoon JY Lee JM Kim-do Y Choi GH Park YN Chung JW Kim EY Park JY Ahn SH Han KH Chon CY A case of embryonal sarcoma of the liver mimicking a hydatid cyst in an adult Gut Liver 2010 4 245 249 10.5009/gnl.2010.4.2.245 20559529 PMC2886927

[B17] Massani M Caratozzolo E Baldessin M Bonariol L Bassi N Hepatic cystic lesion in adult: a challenging diagnosis of undifferentiated primary embryonal sarcoma G Chir 2010 31 225 228 20615364

[B18] Li XW Gong SJ Song WH Zhu JJ Pan CH Wu MC Xu AM Undifferentiated liver embryonal sarcoma in adults: a report of four cases and literature review World J Gastroenterol 2010 16 4725 4732 10.3748/wjg.v16.i37.4725 20872975 PMC2951525

[B19] Gasljevic G Lamovec J Jancar J Undifferentiated (embryonal) liver sarcoma: synchronous and metachronous occurrence with neoplasms other than mesenchymal liver hamartoma Ann Diagn Pathol 2011 15 250 256 10.1016/j.anndiagpath.2010.12.006 21414822

[B20] Kim HH Kim JC Park EK Hur Y Koh YS Cho CK Kim HS Kim HJ Undifferentiated embryonal sarcoma of the liver presenting as a hemorrhagic cystic tumor in an adult Hepatobiliary Pancreat Dis Int 2011 10 657 660 10.1016/S1499-3872(11)60112-4 22146632

[B21] Legou F Ayav A Cahn V Elrifai R Bruot O Régent D Laurent V Radiologic-pathologic comparison of undifferentiated embryonal sarcoma of the liver in a 61-year-old woman Diagn Interv Imaging 2012 93 e208 e211 10.1016/j.diii.2012.01.003 22421287

[B22] Tanaka S Takasawa A Fukasawa Y Hasegawa T Sawada N An undifferentiated embryonal sarcoma of the liver containing adipophilin-positive vesicles in an adult with massive sinusoidal invasion Int J Clin Exp Pathol 2012 5 824 829 23071865 PMC3466983

[B23] Tucker SM Cooper K Brownschidie S Wilcox R Embryonal (undifferentiated) sarcoma of the liver with peripheral angiosarcoma differentiation arising in a mesenchymal hamartoma in an adult patient Int J Surg Pathol 2012 20 297 300 10.1177/1066896911424899 22134632

[B24] Lightfood N Nikfarjam M Embryonal sarcoma of the liver in an adult patient Case Rep Surg 2012 2012 382723 22690347 10.1155/2012/382723 PMC3368299

[B25] Maeshima E Minami Y Sato M Matsuda K Uchiyama K Goda M Ueda H Kida Y Mune M A case of systemic lupus erythematosus with giant hepatic cavernous hemangioma Lupus 2004 13 546 548 10.1191/0961203303lu1040oa 15352428

[B26] Mellemkjaer L Andersen V Linet MS Gridley G Hoover R Olsen JH Non-Hodgkin’s lymphoma and other cancers among a cohort of patients with systemic lupus erythematosus Arthritis Rheum 1997 40 761 768 10.1002/art.1780400424 9125261

[B27] Chen YJ Chang YT Wang CB Wu CY Malignancy in systemic lupus erythematosus: a nationwide cohort study in Taiwan Am J Med 2010 123 1150 e1-e6 21183006 10.1016/j.amjmed.2010.08.006

[B28] Miettinen M Fletcher CDM Kindblom LG Zimmermann A Tsui WMS Mesenchymal tumours of the liver, World health organization classification of tumours of the digestive system 2010 4 Lyon: IARC Press 241 250

[B29] Bisogno G Pilz T Perilongo G Ferrari A Harms D Ninfo V Treuner J Carli M Undifferentiated sarcoma of the liver in childhood: a curable disease Cancer 2002 94 252 257 10.1002/cncr.10191 11815984

[B30] Okajima H Ohya Y Lee KJ Yamamoto H Asonuma K Nagaoki Y Ohama K Korogi M Anan T Hashiyama M Endo F Iyama K Inomata Y Management of undifferentiated sarcoma of the liver including living donor liver transplantation as a backup procedure J Pediatr Surg 2009 44 e33 e38 19231519 10.1016/j.jpedsurg.2008.11.046

[B31] May LT Wang M Albano E Garrington T Dishop M Macy ME Undifferentiated sarcoma of the liver: a single institution experience using a uniform treatment approach J Pediatr Hematol Oncol 2012 34 e114 e116 10.1097/MPH.0b013e3182331fbe 22217489 PMC4131680

